# Squid express conserved ADAR orthologs that possess novel features

**DOI:** 10.3389/fgeed.2023.1181713

**Published:** 2023-06-05

**Authors:** Isabel C. Vallecillo-Viejo, Gjendine Voss, Caroline B. Albertin, Noa Liscovitch-Brauer, Eli Eisenberg, Joshua J. C. Rosenthal

**Affiliations:** ^1^ The Eugene Bell Center, Marine Biological Laboratory, Woods Hole, MA, United States; ^2^ Raymond and Beverly Sackler School of Physics and Astronomy, Tel Aviv University, Tel Aviv, Israel

**Keywords:** squid, Doryteuthis pealeii, cephalopods, RNA editing, ADAR, adenosine deamination, genetic recoding

## Abstract

The coleoid cephalopods display unusually extensive mRNA recoding by adenosine deamination, yet the underlying mechanisms are not well understood. Because the adenosine deaminases that act on RNA (ADAR) enzymes catalyze this form of RNA editing, the structure and function of the cephalopod orthologs may provide clues. Recent genome sequencing projects have provided blueprints for the full complement of coleoid cephalopod ADARs. Previous results from our laboratory have shown that squid express an ADAR2 homolog, with two splice variants named sqADAR2a and sqADAR2b and that these messages are extensively edited. Based on octopus and squid genomes, transcriptomes, and cDNA cloning, we discovered that two additional ADAR homologs are expressed in coleoids. The first is orthologous to vertebrate ADAR1. Unlike other ADAR1s, however, it contains a novel N-terminal domain of 641 aa that is predicted to be disordered, contains 67 phosphorylation motifs, and has an amino acid composition that is unusually high in serines and basic amino acids. mRNAs encoding sqADAR1 are themselves extensively edited. A third ADAR-like enzyme, sqADAR/D-like, which is not orthologous to any of the vertebrate isoforms, is also present. Messages encoding sqADAR/D-like are not edited. Studies using recombinant sqADARs suggest that only sqADAR1 and sqADAR2 are active adenosine deaminases*,* both on perfect duplex dsRNA and on a squid potassium channel mRNA substrate known to be edited *in vivo*. sqADAR/D-like shows no activity on these substrates. Overall, these results reveal some unique features in sqADARs that may contribute to the high-level RNA recoding observed in cephalopods.

## Introduction

The conversion of adenosines to inosines in RNAs is the most frequent form of RNA editing. Because ribosomes read inosine as guanosines, these events can recode mRNAs (Basilio et al., 1962). Historically, protein recoding has been the most intensively studied aspect of RNA editing because the first RNA editing sites that were discovered were recoding events and the functional outcomes of these events were both experimentally tractable and appealing across biological disciplines. Good examples were seminal studies where RNA editing targeted messages encoding ion channel and neurotransmitter receptors, affecting their function and neurophysiology (Sommer et al., 1991; Burns et al., 1997; Bhalla et al., 2004). More recently, however, RNA editing has been shown to alter numerous cellular processes like siRNA processing and substrate recognition (Knight and Bass, 2002; Tonkin and Bass, 2003), message stability and splicing (Rueter et al., 1999; Park et al., 2012; Rieder and Reenan, 2012; Wu et al., 2015) and innate immunity (Mannion et al., 2014; O’Connell et al., 2015). Although it is currently speculated that the ancestral role of RNA editing in vertebrates may have related to innate immunity, this is by no means certain and its role in invertebrates is unknown and may be taxon-specific.

Paradoxically, RNA editing recoding events in mammals are the rarest of all edits. Transcriptome-wide mapping studies have identified millions of RNA editing sites in humans, and tens to hundreds of thousands of sites in other taxa (Porath et al., 2017a; Porath et al., 2017b; Picardi et al., 2017); however, in humans a recent search has uncovered only ∼1,000 recoding sites, ∼200 of which are conserved across mammals (Gabay et al., 2022). Between 100 and 200 recoding sites were identified in zebrafish, ants and bees (Li et al., 2014; Porath et al., 2019; Buchumenski et al., 2021; Duan et al., 2021). In *Drosophila*, recoding is a bit more abundant, with ∼1,000 sites conserved across the taxon (Yu et al., 2016; Duan et al., 2017; Zhang et al., 2017). By far, most editing sites in these species reside in transcribed repetitive elements. Recoding is undoubtedly important in mammals; failure to edit the Q/R site in GluA_2_-mRNAs is lethal (Higuchi et al., 2000). In *Drosophila*, knockout of the single ADAR gene leads to severe neuromuscular defects (Palladino et al., 2000a). Still, most organisms rarely use RNA editing to alter codons.

When compared to other organisms, recoding RNA editing sites are common in cephalopods. In fact, there are ∼57,000 recoding sites in squid (Alon et al., 2015) which occur in the majority of neural transcripts. Two follow-up studies showed that recoding is similarly abundant across coleoids (Liscovitch-Brauer et al., 2017; Shoshan et al., 2021). Many of the recoded mRNAs encode proteins involved in CNS function, as they do in vertebrates and *Drosophila*. Additionally, these editing events can produce functional changes. For instance, editing of potassium channel messages regulates channel kinetics and tetramerization (Rosenthal and Bezanilla, 2002; Liscovitch-Brauer et al., 2017). Editing of squid Na^+^/K^+^ ATPase messages regulates the pump’s turnover rate (Colina et al., 2010). High-level recoding in cephalopods suggests that there is something fundamentally different about their editing machinery. To identify the specific differences, the first features to examine would be the editing enzymes themselves.

A-to-I RNA editing is catalyzed by the ADAR family of enzymes (Bass & Weintraub, 1987; Bass and Weintraub, 1988; Kim et al., 1994a; Melcher et al., 1996a; O’Connell et al., 1998). These enzymes have been most thoroughly studied in mammals and *Drosophila.* Their architecture is similar and consists of two characteristic domains: one or more double-stranded RNA binding motifs (dsRBMs) at their N-terminus followed by a catalytic deaminase domain (DD) at their C-terminus (Kim et al., 1994b; Melcher et al., 1996a). The dsRBMs bind to complex, imperfect dsRNA structures containing mismatches, bulges, and loops, and position the DD near the target adenosine for deamination. In vertebrates, three different ADARs homologs are expressed: ADAR1 and ADAR2, which are functionally active, and ADAR3, which is not (Melcher et al., 1996a; Melcher et al., 1996b; Gerber et al., 1997; Lai et al., 1997; Chen et al., 2000). *Drosophila* expresses a single ADAR isoform that is orthologous to vertebrate ADAR2 (Palladino et al., 2000a). In a previous report, we cloned and characterized a squid ADAR2-ortholog (Palavicini et al., 2009a). The more recently published genome from octopus and two species of squid (Albertin et al., 2015; Belcaid et al., 2019; Albertin et al., 2022), along with multiple transcriptomes (Albertin et al., 2015; Alon et al., 2015; Liscovitch-Brauer et al., 2017; Shoshan et al., 2021; Albertin et al., 2022) allowed us to identify the full complement of cephalopod ADARs. In this work, we describe the unusual structural features, relative abundance, and enzymatic activities of two coleoid cephalopod ADARs and one ADAR/ADAD-like protein.

## Materials and methods

### Cell culture

HEK293T cells were maintained in Dulbecco’s Modified Eagle’s Medium (DMEM) supplemented with 10% fetal bovine serum, 1% penicillin-streptomycin solution, 1 mM sodium pyruvate, and 2 mM glutamine. Cells were seeded in culture dishes and 2 days later were transfected. For transfection, the Effectene Transfection Reagent kit (QIAGEN, Cat No. 301425) was used according to protocol.

### sqADAR1 and sqADAR/D-like cloning

sqADAR1 and sqADAR/D-like sequences were uncovered from a *Doryteuthis pealeii* brain transcriptome that was previously reported (Alon et al., 2015). Using specific primers that were complementary to the sequence surrounding the start and stop codons of each sqADAR, full-length sqADAR1 and sqADAR/D-like were amplified from giant fiber lobe (GFL) cDNA and sequenced to completion. For cloning sqADAR/D-like in the pPICZA FLIS6 expression vector, specific primers were used to amplify the sqADAR/D-like open reading frame (ORF) with SpeI restriction sites. sqADAR1, sqADAR2a, and sqADAR2b were cloned in pcDNA 3.1 (−) for expression in HEK293T cells. These plasmids all had a HIS tag at the N-terminus and a FLAG-tag at the C-terminus and these tags have been shown previously to not affect enzymatic function. FLAG-tagged rADAR2 in pcDNA was provided by Dr. Marie Ohman from Stockholm University. All oligonucleotides used for making the constructs are shown in Supplementary Table S1.

### Purification of sqADAR2 and sqADAR/D-like proteins in *Pichia pastoris*

sqADAR2 and sqADAR/D-like recombinant proteins were purified in *Pichia pastoris* as described previously (Ring et al., 2004; Keegan et al., 2007; Palavicini et al., 2009b).

### Purification of sqADAR1 in HEK293T cells

For purification of sqADAR1, 5 × 10^6^ of HEK293T cells were seeded in three 150 mm dish and 2 days later were transfected with 22 μg plasmid DNA encoding for sqADAR1. 72 h post-transfection, cells were washed with 1X PBS and centrifuged at 100 *g* for 5 min at 4°C. Samples were then lysed with 5 ml buffer (500 mM NaCl, 50 mM HEPES pH 7.4, 1% Triton X-100, and 10% glycerol, supplemented with 5 mM DTT, 0.5 mM PMSF, and 1X Halt Protease Inhibitor cocktail; Thermo Fisher Scientific, Cat No. 78425), incubated for 10 min end-over-end at 4°C, and centrifuged at 2,000 × g for 20 min at 4°C. sqADAR1 protein was then purified with the Ni^2+^-Nitriloacetic acid column (QIAGEN, Cat No. 30310) as described previously (Ring et al., 2004; Keegan et al., 2007; Palavicini et al., 2009b).

### Protein domain identification and phylogenetic tree constructions

We have used a maximum likelihood approach to infer the relationships between domains. The dsRBM and deaminase domains were identified using InterProScan (Jones et al., 2014; Blum et al., 2021) and were aligned with Muscle v5 (Edgar, 2022). Phylogenetic trees were constructed with FastTree2.1 (Price et al., 2010), which employs an (approximately) maximum likelihood approach to infer relationships. The final trees were visualized with Figtree [http://tree.bio.ed.ac.uk/software/figtree/], and each tree was manually rooted to an outgroup. Branch lengths are proportional to sequence divergence, with likelihood of support >75% noted at the nodes.

For the dsRBM and DD alignments, sequences were trimmed according to the complete motifs given in Stefl et al. (2006), Palavicini et al. (2009b), and Chen et al. (2000). Human Staufen1 (Gen-bank AN CAB40082, a.a. 104-173), interferon-induced double-stranded RNA-activated protein kinase like (Genbank AN XP_003441235.2; a.a. 4-71), hADAT (Gene-bank AN NP_036223.2) and hAPOBEC1 (Gen-bank AN P41238.3) were also included in the alignments.

### Self-editing in sqADAR1 and sqADAR/D-like

To determine editing sites in full-length sqADAR1 or sqADAR/D-like mRNA, cDNA synthesized from *D. pealeii* giant fiber lobe (GFL) total RNA (Palavicini et al., 2009a) was amplified and sent for direct sequencing. Genomic DNA amplified from squid GFL (Palavicini et al., 2009a) was used for comparison.

### Non-specific editing assays

Radiolabelled dsRNA substrate synthesis and non-specific editing assays were performed and analyzed as described previously (Palavicini et al., 2009a; Palavicini et al., 2012). In short, the dsRNA substrate was derived from the squid Na^+^ channel GFLN1 (Gen-Bank AN L19979.1; nucleotides 2111–2808) and amplified using primers with the T7 promoter sequence. Recombinant sqADAR proteins (10 nM) were incubated with the radiolabelled dsRNA substrate (0.5p.m.) for 2 h at 35°C in Q140 (10 mM Tris–HCl at pH 7.9, 140 mM KCl, 10 mM NaCl, 20% glycerol) supplemented with 5 mM DTT, 0.5 mM PMSF, 0.5 μg/μl tRNA, and 1X Halt Protease Inhibitor cocktail (Thermo Fisher Scientific, Cat No.78425). For the sqADAR/D-like co-incubation editing assay, the protein concentration was either 10 or 50nM; n = 5.

### Site-specific editing assays

*In vitro* editing assays with the squid potassium channel (sqK_v_1.1) have been described previously (Palavicini et al., 2009a). In short, recombinant proteins (9 nM) were incubated with sqK_v_1.1A mRNA (1 nM) in Q140 Buffer as described above. sqK_v_1.1 mRNA was synthesized using the T7 mScript™ Standard mRNA Production System (CellScript, Cat No. MSC11610). cDNA was then synthesized (Agilent, Cat No. 200436), followed by amplification using Phusion DNA polymerase (New England Biolabs, Cat No. M0530) and sent to Genewiz Inc. for direct sequencing. Only the first 300 nt of the sqK_v_1.1A channel were sequenced, and quantification was done based on C/T dual peak heights. For clarity, sequences shown in the Figures have been reverse complemented and editing shown as A/G dual peak height in electropherograms. Assays were performed in triplicates.

### Relative expression of sqADARs in squid tissue

Three nervous tissues (Optic Lobes, Vertical Lobes and Stellate ganglia) and one non-nervous tissue (Gills) were dissected from 4 adult male specimens of *D. pealeii*. Total RNA was immediately extracted from the fresh tissues using the Trizol reagent (Thermo Fisher Scientific, Cat No. 15596026). 500 ng of total RNA from each sample was then converted into cDNA using Protoscript II Reverse Transcriptase and Oligo dT primers (New England Biolabs, Cat No. M0368).

cDNA from 15 ng total RNA was then quantified using hydrolysis probe-based qPCR with primers specific for sqADAR1, sqADAR2 and sqADAR/D-like (Supplementary Table S2). Primers and Zen/Iowa Black FQ double-quenched FAM-coupled hydrolysis probes were manufactured by Integrated DNA Technologies. The PCR efficiencies of the qPCR assays were tested and in the range of 90%–100% efficiency for all three assays (96% for sqADAR1, 91% for sqADAR2, and 93% for sqADAR/D-like).

PrimeTime Gene Expression Master Mix (Integrated DNA Technologies, Cat No. 1055772) was used for qPCR with 250 nM forward and reverse primers and 150 nM hydrolysis probe. qPCR was performed in triplicates using a BioRad CFX Opus 96 cycler with initial denaturation at 95°C for 3 min, followed by 40 cycles of 5 s at 95°C and 30 s at 60°C.

For absolute quantification, known amounts of gBlocks Gene Fragments (Integrated DNA Technologies) corresponding to the target amplicons were used to prepare a standard curve consisting of 10-fold dilutions between 10^10^ and 10^2^ molecules per reaction. Standard curves were constructed by linear regression and absolute numbers of molecules in the samples were interpolated from the curves.

### Statistical analysis

One-way ANOVA and the Tukey’s test were used for mean comparisons using *p* < 0.05. These tests were used for the data presented in Figure 4.

### Prediction of protein disorder

The IUPred2A program was used to predict the disordered portions of sqADAR1 using the default (long) setting (Abor et al., 2018; Erdős and Dosztányi, 2020).

## Results

### Squid express two ADARs and one ADAR-like protein

**FIGURE 1:**
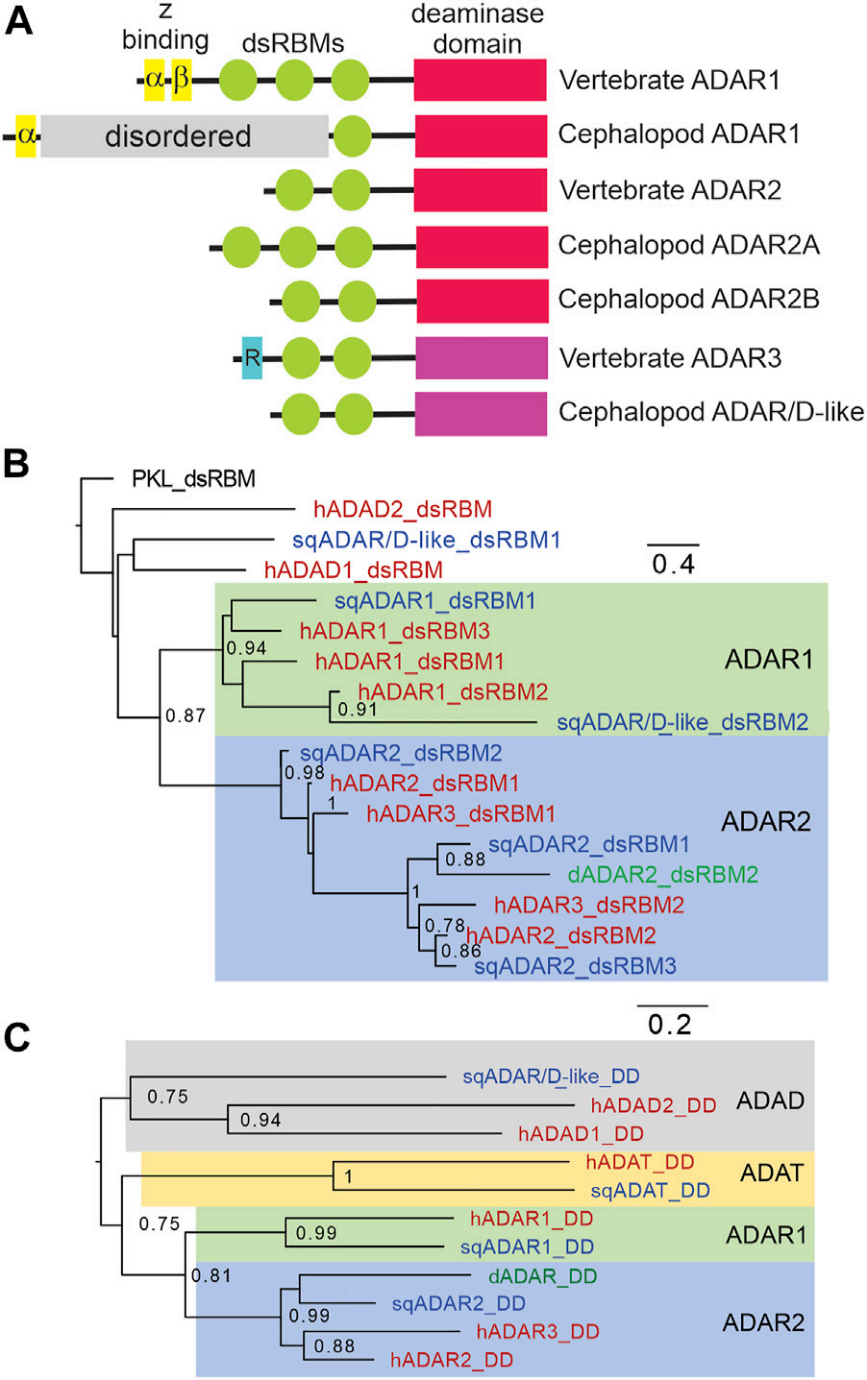
Squid express three ADAR-like enzymes. **(A)** Schematic of vertebrate and squid ADARs and sqADAR/D-like. The ADAR family of enzymes share a similar domain structure, including a DD (shown in red if active or violet if inactive), and a variable number of dsRBMs (green). Z-DNA binding domains (yellow) are unique of ADAR1 isoforms while the R domain (blue), a ssRNA binding domain, is unique to vertebrate ADAR3. sqADAR1 exhibits a novel domain, consisting of a serine rich domain (SRD) which is unique to any known ADAR (grey bar). **(B)** Tree comparing the dsRBMs of squid ADARs and sqADAR/D-like with those of vertebrate ADARs and ADADs. The analysis involved 17 amino acid sequences. All ambiguous positions were removed for each sequence pair. There was a total of 73 positions in the final dataset. The dsRBM of PKL (interferon-induced double-stranded RNA-activated protein kinase like protein) was used as an outgroup **(C)** Tree comparing the DDs of squid ADARs versus vertebrate ADARs. The analysis involved 8 amino acid sequences. All ambiguous positions were removed for each sequence pair.

Taking advantage of published genomes and transcriptomes from squid and octopus (Albertin et al., 2015; Alon et al., 2015; Liscovitch-Brauer et al., 2017; Belcaid et al., 2019; Shoshan et al., 2021; Albertin et al., 2022; Zhang et al., 2023), we searched for cephalopod ADAR homologs (our classifications of squid ADARs are based on trees comparing their dsRBMs and DDs with those of vertebrate ADARs as described later in this section). Blast searches using human ADARs consistently revealed three (Figure 1A). The first is an ADAR1 ortholog. All vertebrate ADAR1s share a common domain structure (Figure 1A): they consist of a Z-DNA binding domain (Z-α), a pseudo Z-DNA binding domain (Z-β), three dsRBMs, and a conserved DD (Patterson and Samuel, 1995; O’Connell et al., 1998). Based on primary sequence comparisons, the sqADAR1 ortholog shares some of these features: a single Z-α domain, a single dsRBM, and an ADAR1-like DD (Figure 1A). Notably, however, it lacks the Z-β domain and two dsRBMs. In their place, it has a long serine-rich domain (SRD; ∼623 amino acids), which contains 67 phosphorylation motifs. Based on blast database searches, we could identify no homologs for this domain.

A second sqADAR was also encountered. It is an ADAR2-ortholog which we have previously cloned and characterized (Palavicini et al., 2009a; Palavicini et al., 2012). Invariably, vertebrate ADAR2s have two dsRBMs at the N-terminus and a conserved DD at the C-terminus (Melcher et al., 1996a; Gerber et al., 1997). *Drosophila* ADAR2 has the same domain architecture (Palladino et al., 2000b). Squid express two splice variants of ADAR2 (sqADAR2a and b). The domain structure of sqADAR2b is canonical, resembling those from vertebrates and *Drosophila*, while sqADAR2a contains an additional dsRBM at the N-terminus. The “extra” dsRBM has been shown to increase sqADAR2a's affinity for RNA (Palavicini et al., 2009b; Palavicini et al., 2012). Interestingly, we found a third protein containing an apparent adenosine deaminase domain. We named it sqADAR/D-like because it cannot be classified as a direct ortholog of any specific ADAR and it is ambiguous whether it is more similar to an ADAR or an ADAD (described in greater detail in the following paragraph). In terms of domain structure, like vertebrate ADAR2 and ADAR3 it has two dsRBMs and a DD. The complete sequences for all ADARs were amplified and cloned from nervous system cDNA and verified by Sanger sequencing. The sequences for sqADAR1 and sqADAR/D-like are given in Supplementary Figure S1 and the sequences for sqADAR2a and sqADAR2b were published previously (Palavicini et al., 2009a).

Squid ADARs were classified based on trees using their dsRBMs or DDs. The dsRBM of sqADAR1 groups into a general ADAR1 cluster (Figure 1B). For sqADAR2, the dsRBMs cluster into a general human ADAR2/ADAR3 cluster. The first dsRBM of sqADAR2 (dsRBM1; the “extra” dsRBM specific to sqADAR2A) groups with dsRBM2 of hADAR2, *Drosophila* ADAR2 and hADAR3. dsRBM2 of sqADAR2 groups with dsRBM1 from hADAR2 and *Drosophila* ADAR2 and dsRBM3 of sqADAR2 groups with dsRBM2 of hADAR2 and *Drosophila* ADAR2. Interestingly the dsRBM1 of sqADAR/D-like does not group with ADAR dsRBMs, nor with high confidence to the ADAD dsRBMs. Its second dsRBM groups with ADAR1s. When we look at the DDs (Figure 1C), sqADAR1’s DD is clearly an ADAR1-type and sqADAR2’s is clearly an ADAR2-type. That of sqADAR/D-like groups with low confidence to those of the human ADADs over those of ADARs.

### Squid *ADAR* gene architecture

**FIGURE 2:**
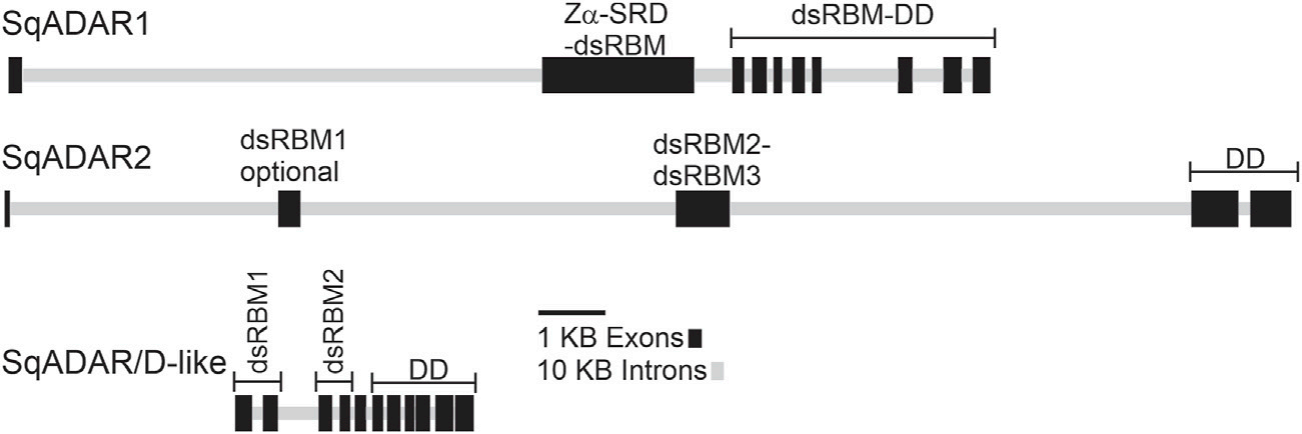
sqADAR1, sqADAR2, and sqADAR/D-like genes. Schematic representation of squid ADAR1, ADAR2, and ADAR/D-like genes. Exons are represented by dark grey boxes and introns are represented by light gray line. The domain encoded within each exon is also indicated. dsRBM = double-stranded RNA binding motif, SRD = serine rich domain, and DD = deaminase domain.

The structures of the squid ADAR and ADAR/D-like genes were determined by comparing our cDNA sequences for sqADAR1, sqADAR2 and sqADAR/D-like to the gene models predicted from the *D. pealeii* genome (Albertin et al., 2022) (Figure 2). The genes vary greatly. The coding regions of sqADAR1 and sqADAR2 are large, spanning ∼120 KB and ∼175KB, respectively. At ∼15 kB, the coding region of sqADAR/D-like is relatively small. sqADAR1 has 10 coding region exons. Interestingly, exon 2 encodes the Z-α domain, the SRD and the lone dsRBM, indicating that the SRD, which is a novel structure (discussed in the following section), did not arise by an independent exonization event. The DD of sqADAR1 is encoded by 8 different exons. At ∼75 kB, the first intron of sqADAR1 is large. With 5 coding region exons, sqADAR2 is simpler. The optional first dsRBM is encoded by exon 2, the second and third dsRBMs are encoded by exon 3, and the DD is encoded by exons 4 and 5. The first three introns of sqADAR2 are large, from ∼40 to 65 KB each. Although relatively small, the coding region of sqADAR/D-like is highly fragmented across 11 exons.

### Unusual features of squid ADARs

**FIGURE 3:**
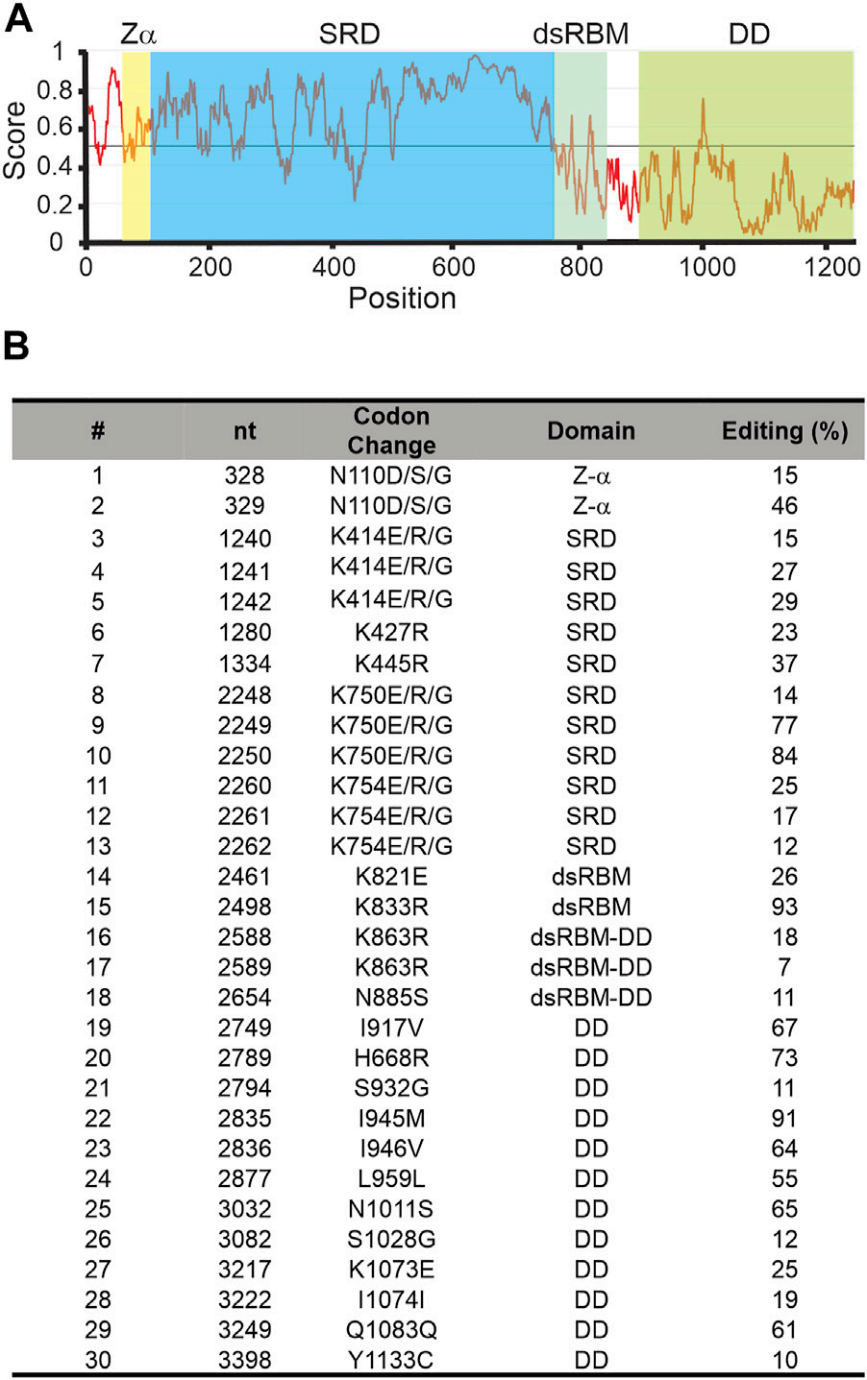
Squid ADAR1 sequence shows sequence disorder and extensive self-editing. **(A)** Sequence-based prediction of disorder within sqADAR1 using IUPred2A using default parameters. **(B)** Editing in sqADAR1 mRNA. Based on dual peaks in direct sequencing electropherograms from cDNA synthesized from squid GFL, 30 editing sites have been identified in sqADAR1 mRNA. Four sites (N110, K414, K750, and K754) occur within the same codon, generating multiple possibilities. Editing percentages were estimated from electropherogram peaks. dsRBM = double-stranded RNA binding motif, SRD = serine-rich domain, and DD = deaminase domain.

Of the 3 squid ADARs, sqADAR1 has the most unusual features. Besides lacking 2 of the 3 dsRBDs and the Z-β domain normally found in ADAR1s, it has a SRD (Figure 1A). Based on BLAST database searches, outside of the coleoid clade, no other taxa express an ADAR1 with a similar domain. In fact, there seem to be no homologous domains to the SRD in any protein. At 623 amino acids, the SRD is large and occupies most of the N-terminus. Its predicted isoelectric point is 9.39 and at pH 7, its charge is 20.22. Approximately 20% of its amino acids are serines. Additionally, it is rich in lysines and arginines, comprising almost 12% of the domain. Taken together, the enrichment in these amino acids creates 67 potential phosphorylation sites. A striking feature of the SRD is that it is predicted to be highly disordered (Figure 3A; see methods). The domain’s function is entirely unknown.

Another intriguing feature of sqADAR1 is that its own transcripts are extensively edited. We amplified the entire ORF of sqADAR1 in overlapping PCR amplicons using giant fiber lobe neurons (GFL; cell bodies of the giant axon) cDNA as a template. Sanger sequencing revealed 30 editing sites (Figure 3B). These sites occur within all domains of sqADAR1 and are edited to different extents. Two sites are found in the Z-α domain, within the same codon. They can generate 3 alternative amino acids (N110D, S, or G). There are 11 sites within 5 codons in the SRD. Interestingly, all editing sites within the SRD recode a lysine (AAA) residue. Three of these codons (K414, K750, and K754) are edited at all three positions, leading to outcomes K, E, R or G. These changes may lead to a possible charge reversal (K > E), a side-chain erasure (K > G) or a minimal change (K > R). There are 2 sites in the single dsRBM, 4 in the linker between the dsRBM and the DD, and 12 in the DD, 9 of which recode. Due to their locations, some editing events are worth highlighting (Supplementary Figure 1): N110 is important for Z-DNA binding (Schwartz et al., 1999); K833, within the dsRBM, is important for RNA binding (Stefl et al., 2006); and K1073, within the DD, makes direct contact with the IP_6_ molecule around which the domain folds (Macbeth et al., 2005). As previously published (Palavicini et al., 2009b), sqADAR2 messages are also highly edited, although to a lesser extent than sqADAR1 messages. Unlike sqADAR1 or sqADAR2, no editing sites were found in sqADAR/D-like mRNAs.

### sqADAR1 is functional while sqADAR/D-like is not

**FIGURE 4:**
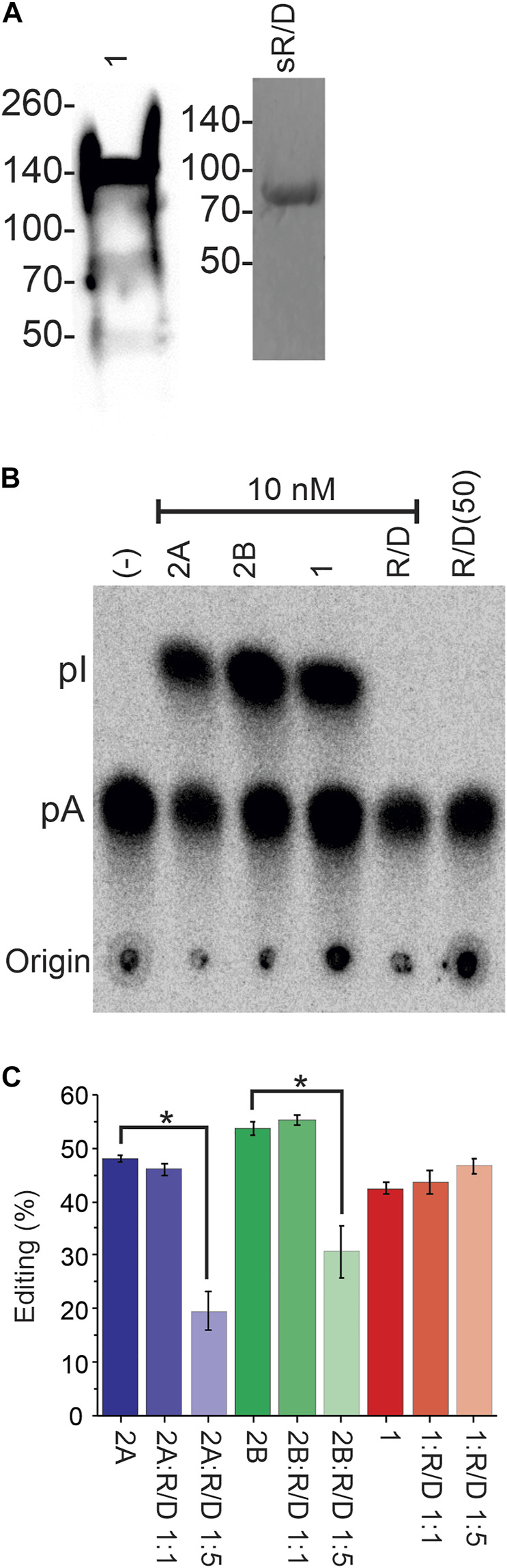
Recombinant sqADAR1 is active while sqADAR/D-like is not. **(A)** Purification of sqADAR1 and sqADAR/D-like. (left panel) Western blot of sqADAR1 purification from HEK293T cells. Fraction eluted from a Ni^2+^-NTA column is shown. Lysates were run on a 4%–20% gradient gel and transferred onto PVDF membranes. Membranes were probed with a primary antibody α-FLAG at 1:3000 followed by a stabilized peroxidase conjugated goat anti-mouse (1:5,000) secondary antibody. The predicted size for sqADAR1 is ∼137 kDa. Note that it is susceptible to degradation. Positions of protein standards are indicated on the left. (Right panel) SDS-PAGE gel of the purification of sqADAR/D-like from *Pichia pastoris*. Fraction eluted from anti-FLAG column was electrophoresed on a 4%–20% gradient gel and stained with Coomassie Blue. The predicted size for sqADAR/D-like is 71 kDa. Again, the position of protein standards are indicated on the left. **(B)** Example of TLC analysis of non-specific ADAR activity assay with radiolabelled, perfect-duplex dsRNA. Recombinant sqADARs were incubated with radiolabelled dsRNA, digested with P1 nuclease, followed by TLC separation. **(C)** TLC analysis from editing assays with radiolabelled dsRNA incubated with either sqADAR2a, sqADAR2b, or sqADAR1 alone or with 1:1 or 1:5 ratio of sqADAR/D-like. A to I conversion was calculated from Phosphorimager scans of the TLC plates. n = 5 ± s.e.m (ii). Asterisk indicates significance with *p* < 0.05. ​1 = sqADAR1, 2A = sqADAR2A, 2B = sqADAR2B and R/D = sqADAR/D-like.

We next explored whether the sqADARs are catalytically active. In mammals, both ADAR1 and ADAR2 can convert adenosines to inosines in RNA, but ADAR3 cannot (Kim et al., 1994a; Kim et al., 1994b; O'Connell & Keller, 1994; Gerber et al., 1997; Lai et al., 1997; O’Connell et al., 1998; Chen et al., 2000). In previous studies, we purified sqADAR2a and sqADAR2b from *P. pastoris* and showed that both forms are active (Palavicini et al., 2009b; Palavicini et al., 2012). Here, we tried to purify sqADAR1 from *P. pastoris* but were unsuccessful (ADAR1s are notoriously difficult to purify from heterologous systems). Small amounts of sqADAR1, however, could be purified from transiently transfected HEK293T cells (Figure 4A). sqADAR/D-like, on the other hand, was readily purified from *P. pastoris* following the same approach that was used to purify sqADAR2 (Palavicini et al., 2009b; Palavicini et al., 2012). Thus, even though they could not be purified from the same system, we were in a position to test the functionality of both sqADAR1 and sqADAR/D-like. It should be noted that both Pichia pastoris and HEK-293 cells produce functional sqADAR2 (Palavicini et al., 2009b; Palavicini et al., 2012; Vallecillo-Viejo et al., 2020).

We first tested recombinant sqADAR1 and sqADAR/D-like’s ability to promiscuously edit A’s in a long perfect-duplex dsRNA using a non-specific assay as previously described (Palavicini et al., 2009b; Palavicini et al., 2012). In brief, recombinant enzyme is incubated with a 711 bp perfect RNA duplex that contains α^32^P-labelled adenosines. If the enzyme is active, some adenosines will be converted to inosine. After incubation, dsRNA substrate is digested into 5′-nucleoside monophosphates, and the labelled nucleosides are separated by thin layer chromatography (TLC). As a positive control, sqADAR2a and sqADAR2b gave robust catalytic activity converting 48% ± 2% and 53% ± 3% of the A’s→I’s, respectively (Figure 4B). sqADAR1 was active as well, converting 43% ± 1% of the A’s→I’s. sqADAR/D-like, on the other hand, showed no activity, even when added in a 5-fold excess. We next tested whether mixing sqADAR/D-like with sqADAR1, sqADAR2a, or sqADAR2b would generate synergistic or antagonistic effects. Interestingly, sqADAR/D-like, when added in excess, could block sqADAR2a and sqADAR2b activity, but had no effect on sqADAR1 under the same conditions (Figure 4C). Taken together, these results indicate that sqADAR1 and sqADAR2 are active, but sqADAR/D-like is not, although it might be able to regulate the activity of sqADAR2s.

**FIGURE 5:**
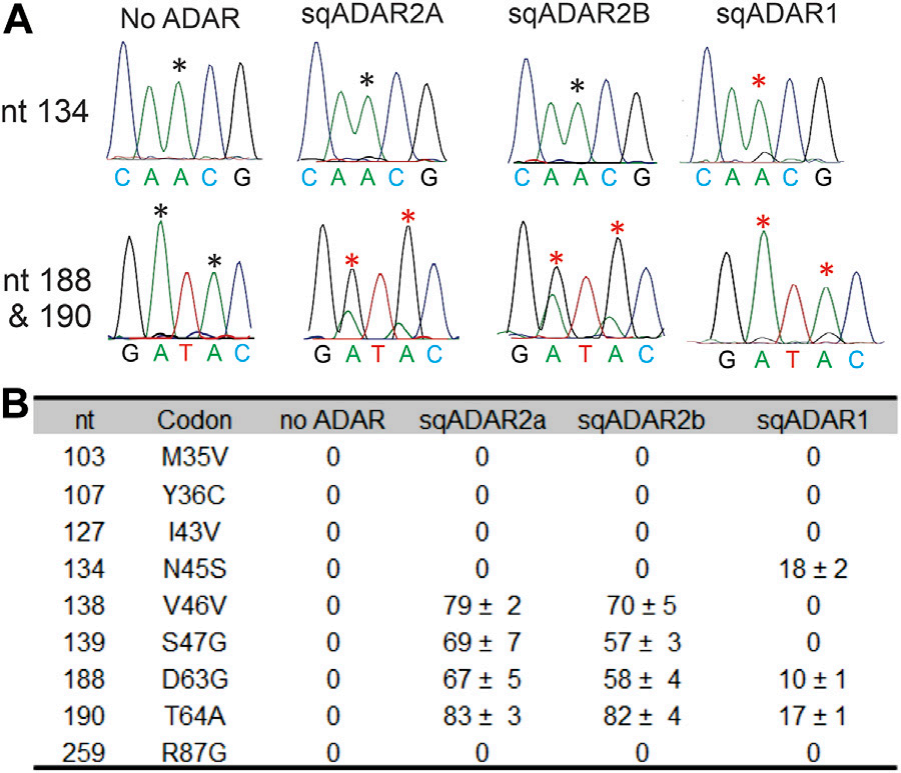
sqADAR1 edits sqKv1.1 mRNA. **(A)** Electropherograms from RT-PCR products of *in vitro* editing assays with sqKv1.1 mRNA. Asterisks indicate the edited adenosines. **(B)** Table summarizing editing percentages for specific editing sites identified in the first 300 bp of sqKv1.1. Nt = the position of the edited adenosine in the sqKv1.1 ORF. Codon refers to the specific codon change after editing; n = 3 ± s.e.m.

We next tested sqADAR1’s ability to edit a specific target *in vitro*. As a substrate for these experiments, we used a portion of the mRNA encoding the sqK_v_1.1 channel, as it is known to be heavily edited *in vivo* (Rosenthal and Bezanilla, 2002; Alon et al., 2015; Liscovitch-Brauer et al., 2017) and can be edited at some sites *in vitro* using recombinant sqADAR2 (Palavicini et al., 2009b; Palavicini et al., 2012)*.* We incubated recombinant sqADAR1 with sqKv1.1 mRNA and looked for editing by RT-PCR and Sanger sequencing, using sqADAR2a and sqADAR2b as controls. We focused on the first 300 nt because this region contains 9 sites that are edited *in vivo*. Examples of electropherograms at positions 134, 188, and 190 are shown in Figure 5A. Positions 188 and 190 are edited by sqADAR2a, sqADAR2b, and sqADAR1, although sqADAR1 edited them to a lower extent. Position 134 was only edited by sqADAR1, as no editing was seen for sqADAR2a or sqADAR2b. In all, sqADAR2a and sqADAR2b edited four sites (V46V, S47G, D63G, and T64A; Figure 5B). sqADAR1 edited two of the four (D63G and T64A) and an additional site not edited by sqADAR2a or sqADAR2b (N45S). Thus within sqK_v_1 messages, sqADARs have overlapping but distinct specificities, and are similar to vertebrate ADAR1 and ADAR2 in this regard (Lehmann and Bass, 2000).

### Relative expression of sqADARs in squid tissue

**FIGURE 6:**
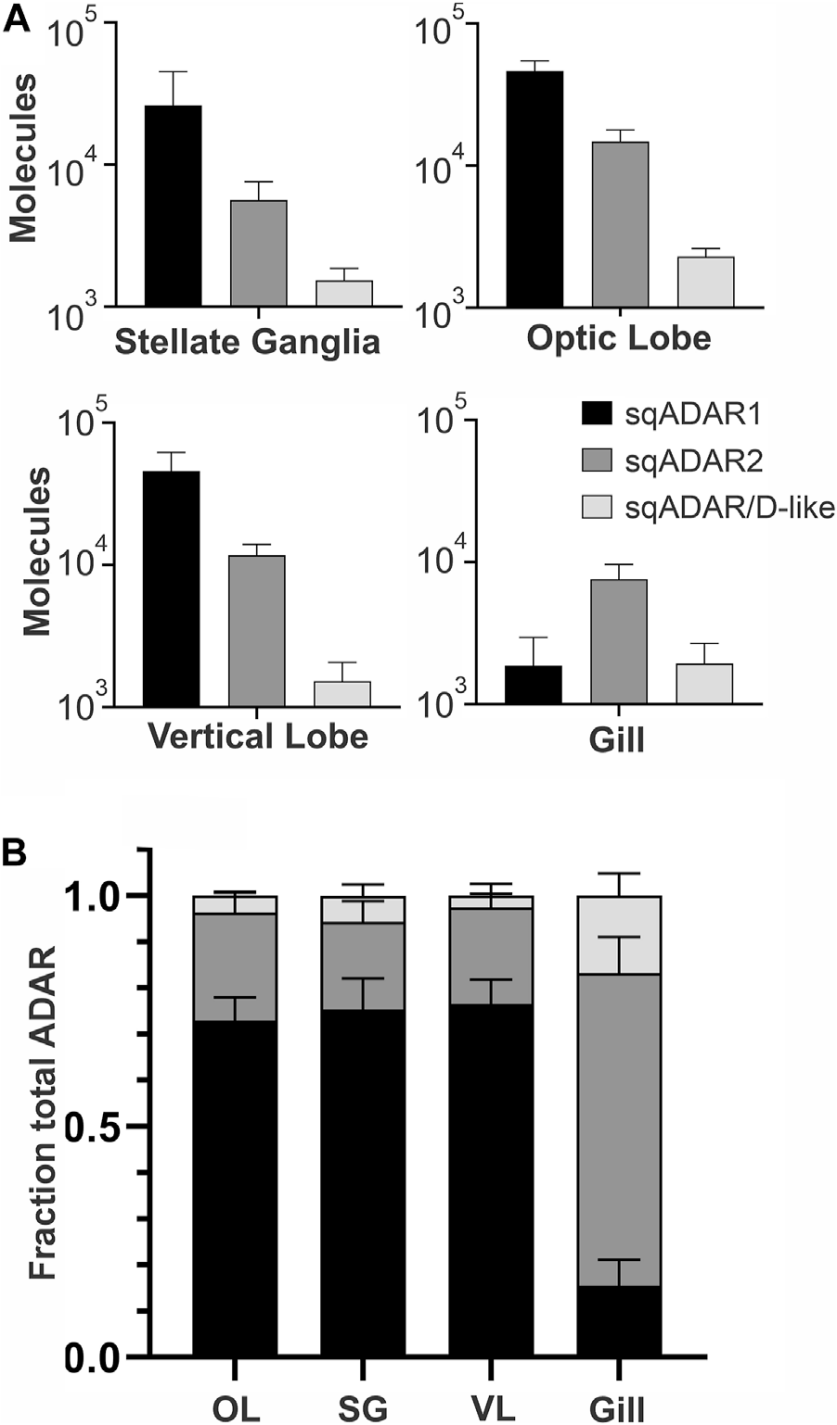
Relative expression of sqADARs in different tissues. Relative expression of all sqADARs was determined by qPCR in both nervous and non-nervous tissue (see methods). **(A)** The absolute number of ADAR molecules within a sample was extrapolated from a standard curve generated using serial dilutions of synthetic gene fragments encoding the amplicons. **(B)** The total fraction of each ADAR from the pool of total ADAR was derived from the data in **(A)** n = 4 and error bars are SD.

We next examined the relative expression of the three sqADAR enzymes in three neural (optic lobe, stellate ganglia and vertical lobe) and one non-neural (gill) tissues. This was accomplished using qPCR. Specific primer pairs were designed for each ADAR (Supplementary Table S2) and tested to ensure that they amplified with suitable efficiencies. Fluorescence accumulation during reactions was monitored using Zen/Iowa Black FQ double-quenched FAM-coupled hydrolysis probes (Supplementary Table S2). A standard curve was constructed for each ADAR using a geneblock DNA fragment corresponding to the target amplicon and was used to estimate the number of ADAR cDNA molecules generated from a defined quantity of total RNA from each tissue. Given the inherent variations between RNA preps and reverse transcription, ADAR expression between samples cannot be accurately compared between tissue samples without normalization to housekeeping genes. However, the relative expression of each ADAR within a sample can be. Figure 6A shows that ADAR1 is the mostly highly expressed ADAR in nervous tissues, followed by ADAR2 and then ADAR/D-like. In the gills, ADAR2 is the most highly expressed, followed by ADAR1 and ADAR/D-like at similar levels. Within the nervous system, ADAR1 makes up ∼70% of the total ADAR message and ADAR2 makes up ∼ 25% of it (Figure 6B). ADAR/D-like is expressed at low levels. Conversely, in the gill ADAR2 makes up ∼70% of the total ADAR message. It is interesting to note that in vertebrates ADAR2 is generally more highly expressed in the nervous system than ADAR1.

## Discussion

The high-level recoding by RNA editing in the coleoid cephalopods appears unique, yet the underlying mechanisms are not well understood. Data presented in this study provide a catalogue of candidate features that may contribute to extensive editing. We also provide a complete list of the complement of functional sqADARs based on transcriptomics, genomics, functional assays, and expression patterns. As vertebrates, squid express two active ADARs and a catalytically inactive one, and the two active ones are orthologous between the clades. Despite these similarities, the two active squid ADARs possess unique domains. Particularly striking are the SRD of sqADAR1, the extra dsRBM of sqADAR2 and the fact that the mRNAs encoding both are themselves extensively edited.

Of the two active sqADARs, sqADAR1 is the more divergent from the vertebrate orthologs. First of all, it is missing the Z-β domain and two of the three dsRBMs. On top of this, the SRD is a truly unique feature for an ADAR and draws one to speculate about its purpose. The SRD is very positively charged at pH 7, which could promote non-specific binding to the negatively charged RNA backbone. This RNA binding could be further regulated via phosphorylation. In the SRD there are 67 potential phosphorylation sites, and the addition of these negative charges would serve to reduce RNA binding, perhaps in a graded manner. Another level of control could be by editing adenosine residues within the sqADAR1 mRNA itself. In the serine-rich region there are a total of 11 editing sites within 5 lysine codons, recoding the position to an E, R or G. These changes would lower the pKa and thus affect RNA binding. Finally, the SRD is predicted to be intrinsically disordered; thus it may serve to promote phase partitioning, or promote sqADAR1’s association with partitioned RNA in granules. The relationship between edited RNA and RNA phase separation/RNA granules is unexplored.

There are many editing sites in the sqADAR1 messages, with many in areas outside of the SRD, and some have the obvious potential to regulate activity. For example, codon N110 can be edited to D, S or G and this residue has been shown to be important for Z-DNA binding (Schwartz et al., 1999). In addition K833 can be edited to R within the dsRBM, and this position has been shown to alter recognition and binding of dsRNA (Stefl et al., 2006). Finally, K1073 can be edited to E within the DD. Structural data shows that this position helps to coordinate IP_6_ binding (Macbeth et al., 2005) thus this edit may affect folding or catalysis. Taken together, the SRD, the large number of phosphorylation motifs and the multiple editing sites within sqADAR1 mRNAs create an enormous potential for structural diversity and one would speculate that sqADAR1’s activity is tightly controlled.

The expression pattern of sqADARs across tissues might provide clues on their activities. qPCR analysis revealed that sqADAR1 is the most highly expressed isoform in the nervous system and sqADAR2 is the predominant ADAR in the gill. Message recoding is many times higher in the nervous system than in other tissues, however stochastic editing in non-coding regions is spread uniformly across tissues (Liscovitch-Brauer et al., 2017; Albertin et al., 2022). Accordingly, sqADAR1 may catalyze the recoding in the nervous system and sqADAR2 catalyze the pan-tissue editing. Our *in vitro* editing results on sqKv1 messages, however, do not agree with this idea.

sqADAR2 edits 4 of the 9 sites in our substrate that are known to be edited *in vivo* (Rosenthal and Bezanilla, 2002) while sqADAR1 edits 2 of these sites, and one additional one (Figure 5). At the common sites, sqADAR2 edits more extensively than sqADAR1. Notably, 4 of the 9 sites found *in vivo* are not edited by either sqADAR in our *in vitro* assay. The inability to reproduce all naturally occurring editing sites *in vitro* could be due to the fact that the entire structure required for editing is not encoded in this substrate. For instance, the sqK_v_1.1 pre-mRNA contains a very large intron at the beginning of the pre-mRNA coding sequence and none of the intronic sequence is included in our substrate. Another possibility is that there are other accessory proteins or RNAs in squid that influence editing site selection.

Interestingly, both cephalopods and vertebrates appear to require a catalytically inactive ADAR-like protein, at least in terms of adenosine deamination. The same cannot be said for *Drosophila,* whose genome encodes a single ADAR, a fully-active ADAR2 ortholog (Palladino et al., 2000b). sqADAR/D-like has a domain structure much like vertebrate ADAR3 (2 dsRBMs and a DD), however the sequence encoding these elements is no closer to vertebrate ADARs than ADADs. In this study, we show sqADAR/D-like has no deamination activity on perfectly duplexed dsRNA. In addition, unlike the sqADARs, its own mRNA does not appear to be edited. sqADAR/D-like’s lack of activity is supported by the fact that key residues known to be involved with catalysis are mutated (Table 1). These include two of the three positions that coordinate a zinc ion at the active site (C451S and C516A) and one involved in proton shuttling (E396A; Macbeth et al., 2005). Residues within the DD that make direct contact with dsRNA (Matthews et al., 2016) are also missing. In fact, judging from the mutations, sqADAR/D-like appears more dysfunctional than human ADAR3. Even though inactive, we have shown that sqADAR/D-like is able to inhibit sqADAR2-mediated RNA editing, but not sqADAR1 *in vitro*. In vertebrates, hADAR3 has also been shown to inhibit ADAR2 activity (Chen et al., 2000). sqADAR/D-like does not appear to be cephalopod-specific as BLAST searches uncover similarities to the recently described ADAD-like molecules in other mollusks (Zhang et al., 2023). ADADs act as RNA-binding molecules found to be essential for spermatogenesis in mammals (Schumacher et al., 1995; Snyder et al., 2020; Dai et al., 2023). The function of ADAD-like molecules in mollusks is unknown.

**TABLE 1:** Critical DD residues for different ADARs. The Zn^2+^ chelating residues, H^+^-shuttle, and direct IP_6_ binding residues important for deamination are shown. Residues that indirectly bind to IP_6_ via a water molecule are not included. Important residues that make contact with dsRNA are also indicated. Residues that are not conserved with hADAR2 are shown in bold.

Function	HADAR2	sqADAR2a	sqADAR1	sqADAR/D-like	HADAR3
Zn^2+^ Coord	C451	C516	C996	**S368**	C490
Zn^2+^ Coord	C516	C580	C1070	**A443**	C555
Zn^2+^ Coord	H394	H458	H942	H311	H432
H^+^ Shuttle	E396	E460	E944	**A313**	E434
IP_6_ Binding	R400	R464	R948	R317	R438
IP_6_ Binding	R401	R465	R949	R318	R439
IP_6_ Binding	K629	K693	**S1189**	**S568**	K668
IP_6_ Binding	Y658	Y722	Y1218	Y593	Y696
IP_6_ Binding	K662	K726	K1222	K597	K700
IP_6_ Binding	S531	**A595**	**A1085**	**A458**	**A570**
IP_6_ Binding	Y668	Y732	**F1228**	Y603	Y706
IP_6_ Binding	K672	K736	K1232	K607	K710
IP_6_ Binding	R522	R586	R1076	**K449**	R561
IP_6_ Binding	W687	W751	W1247	W622	W725
IP_6_ Binding	K690	K754	K1250	K625	K728
IP_6_ Binding	K519	K583	K1073	K446	K558

Bold values show the residues that are not conserved from hADAR2 (says so in the legend).

In summary, the molecular underpinnings of squid ADARs are complex. The gene complement is remarkably similar to that of vertebrates: an ADAR1 ortholog, an ADAR2 ortholog and a third protein that may play a similar role as ADAR3, ADAD1 or ADAD2, or perhaps a wholly different role. Many of the features within these genes, however, appear to be unique for cephalopods. Of particular interest are the SRD of sqADAR1, the extra dsRBM of sqADAR2, and the extensive self-editing of the transcripts for both. Recent advances on the use of CRISPR-Cas9 mediated gene knockouts in cephalopods will enable us to explore these features more thoroughly with the goal of better understanding the mechanisms underlying high-level RNA recoding in the coleoid cephalopods.

## Data Availability

The original contributions presented in the study are included in the article/Supplementary Materials, further inquiries can be directed to the corresponding author.
