# Supplementary material for: Squid express conserved ADAR orthologs that possess novel features
Source: Front Genome Ed. 2023 Jun 5;5:1181713. doi: 10.3389/fgeed.2023.1181713 (PMC10278661; doi:10.3389/fgeed.2023.1181713)
Supplement: Supplementary file 1 [file Table1.docx]

Supplementary Material

**Squid express conserved ADAR orthologs that possess novel features**

Isabel Vallecillo-Viejo^1,2^, Gjendine Voss^1^, Caroline Albertin^1^, Noa Liscovitch-Brauer^3^, Eli Eisenberg^3^, Joshua Rosenthal^1*^

^1^ The Eugene Bell Center, Marine Biological Laboratory, Woods Hole, MA, USA

^2^ Current address: Department of Biomedical Engineering and Department of Pediatrics, Vanderbilt University and Vanderbilt University Medical Center, Nashville, TN, USA

^3^ Raymond and Beverly Sackler School of Physics and Astronomy, Tel Aviv University, Tel Aviv, Israel

*Correspondence:

Joshua Rosenthal

jrosenthal@mbl.edu

**Supplementary Table 1.** Oligos used in this study for cloning.

|  | **Name** | **Sequence** |
| --- | --- | --- |
| sqADAR1 | Forward primer | 5′-CCTGAGAACCTTGCTGGGAG-3′ |
|  | Reverse primer | 5′-GACACTGTTGCCTTCCAAACTC-3′ |
|  | Hydrolysis probe | 5′-(6-FAM)-TGTTGGAAC-(ZEN)-TGGTAACCGCTGTTTAACCGG-(Iowa Black FQ)-3′ |
| sqADAR2 | Forward primer | 5′-GAAACAGGCCAAAGCTAGAGC-3′ |
|  | Reverse primer | 5′-CTTCTCGAGTACTGGCTTG-3′ |
|  | Hydrolysis probe | 5′-(6-FAM)-CTGGTTGAA-(ZEN)-GGCCAGGTGGGAAAAGTTCC-(Iowa Black FQ)-3′ |
| sqADAR/D-like | Forward primer | 5′-CATGCTGACCACGGATGGAAG-3′ |
|  | Reverse primer | 5′-TTTCACTTTTAGGGTTCTCTCATTAGGG-3′ |
|  | Hydrolysis probe | 5′-(6-FAM)-CAATTGTCA-(ZEN)-GGTATCGACGGAAAGTTCTTCTTGC-(Iowa Black FQ)-3′ |

Supplementary Table 2. Oligos used in this study for qPCR


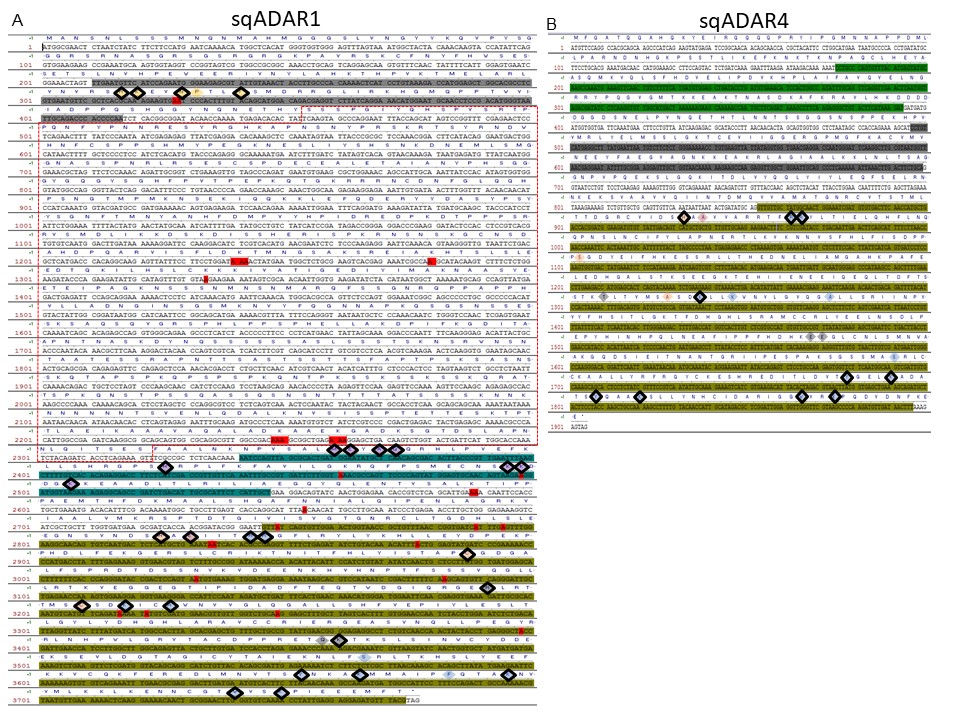


**Supplementary Figure 1.** Open reading frames for sqADAR1 and sqADAR/D-like. A) ORF for sqADAR1. Domains are colored as follows: Z-α (grey), dsRBM (teal), and deaminase domain (dark yellow). The serine-rich region is enclosed in a dashed red box. Edited adenosines are highlighted in red. Based on structural data, amino acids that make direct contact with Z-DNA (Z-α) are enclosed in yellow diamonds and those that make direct contact with RNA (dsRBM) are in purple. A black border indicates that the position is conserved between squid and human ADAR1. Blue diamonds indicate positions that make direct contact with IP_6_ in human ADAR1, green diamonds represent the three Zn^2+^ chelating residues and the red diamond represents the proton-shuttling residue important for deamination. Grey diamonds enclose the deaminase domain residues that make contact with dsRNA. Again, a black border indicates conservation. B) ORF for sqADAR/D-like. Green represents dsRBM1 and grey represents dsRBM2. The deaminase domain is highlighted in dark yellow. Blue diamonds indicate positions that make direct contact with IP_6_, green diamonds represent the three Zn^2+^ chelating residues and the red diamond represents the proton-shuttling residue important for deamination. Grey diamonds represent the deaminase domain residues that make contact with dsRNA. A black border indicates conservation.
