# Supplementary figures and images for: Squid express conserved ADAR orthologs that possess novel features
Source: Front Genome Ed. 2023 Jun 5;5:1181713. doi: 10.3389/fgeed.2023.1181713 (PMC10278661; doi:10.3389/fgeed.2023.1181713)

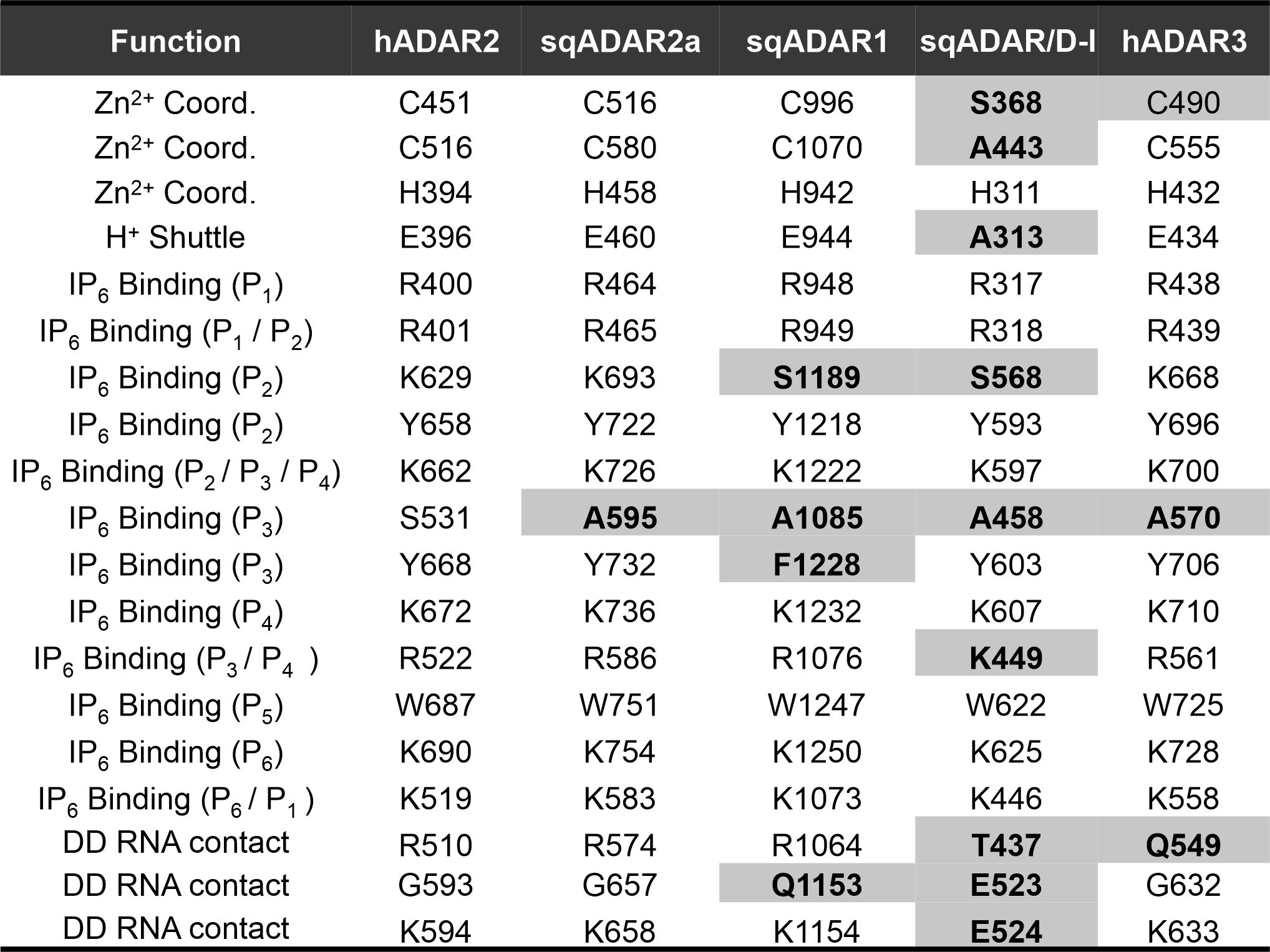

Supplement: Supplementary file 2 [file Image1.JPEG]
