# Supplementary material for: Squid express conserved ADAR orthologs that possess novel features
Source: Front Genome Ed. 2023 Jun 5;5:1181713. doi: 10.3389/fgeed.2023.1181713 (PMC10278661; doi:10.3389/fgeed.2023.1181713)
Supplement: Supplementary file 3 [file DataSheet1.PDF]

Function	hADAR2	sqADAR2a	sqADAR1	sqADAR/D-I	hADAR3
Zn^{2+} Coord.	C451	C516	C996	**S368**	C490
Zn^{2+} Coord.	C516	C580	C1070	**A443**	C555
Zn^{2+} Coord.	H394	H458	H942	H311	H432
H^+ Shuttle	E396	E460	E944	**A313**	E434
IP_6 Binding (P_1)	R400	R464	R948	R317	R438
IP_6 Binding (P_1 / P_2)	R401	R465	R949	R318	R439
IP_6 Binding (P_2)	K629	K693	**S1189**	**S568**	K668
IP_6 Binding (P_2)	Y658	Y722	Y1218	Y593	Y696
IP_6 Binding (P_2 / P_3 / P_4)	K662	K726	K1222	K597	K700
IP_6 Binding (P_3)	S531	**A595**	**A1085**	**A458**	**A570**
IP_6 Binding (P_3)	Y668	Y732	**F1228**	Y603	Y706
IP_6 Binding (P_4)	K672	K736	K1232	K607	K710
IP_6 Binding (P_3 / P_4)	R522	R586	R1076	**K449**	R561
IP_6 Binding (P_5)	W687	W751	W1247	W622	W725
IP_6 Binding (P_6)	K690	K754	K1250	K625	K728
IP_6 Binding (P_6 / P_1)	K519	K583	K1073	K446	K558
DD RNA contact	R510	R574	R1064	**T437**	**Q549**
DD RNA contact	G593	G657	**Q1153**	**E523**	G632
DD RNA contact	K594	K658	K1154	**E524**	K633
